# Evaluation of Serum Leucine-Rich Alpha-2 Glycoprotein as a New Inflammatory Biomarker of Inflammatory Bowel Disease

**DOI:** 10.1155/2021/8825374

**Published:** 2021-02-01

**Authors:** Tetsuhiro Yoshimura, Keiichi Mitsuyama, Ryosuke Sakemi, Hidetoshi Takedatsu, Shinichiro Yoshioka, Kotaro Kuwaki, Atsushi Mori, Shuhei Fukunaga, Toshihiro Araki, Masaru Morita, Kozo Tsuruta, Hiroshi Yamasaki, Takuji Torimura

**Affiliations:** ^1^Division of Gastroenterology, Department of Medicine, Kurume University School of Medicine, 67 Asahi-machi, Kurume 830-0011, Japan; ^2^Inflammatory Bowel Disease Center, Kurume University Hospital, 67 Asahi-machi, Kurume 830-0011, Japan; ^3^Department of Gastroenterology, Tobata Kyoritsu Hospital, Kitakyushu, Japan

## Abstract

Studies on serum leucine-rich alpha-2 glycoprotein (LRG) in inflammatory bowel disease (IBD), including ulcerative colitis (UC) and Crohn's disease (CD), are scarce; the methods for estimating disease activity are less established, particularly for CD. This study is aimed at evaluating the utility of serum LRG as a potential inflammatory marker for IBD and to investigate the LRG gene expression in peripheral blood mononuclear cells (PBMCs) as a possible source of serum LRG. Overall, 98 patients with UC and 96 patients with CD were prospectively enrolled and clinically evaluated; 92 age-matched individuals served as the healthy controls. The blood samples were analyzed for serum LRG levels and routine laboratory parameters. Disease activity was assessed clinically and endoscopically. Finally, LRG gene expression in the PBMCs from a different cohort (41 patients with UC, 34 patients with CD, and 30 healthy controls) was examined. The serum LRG levels were higher during active disease than during inactive disease; additionally, serum LRG levels were positively correlated with clinical disease activity, C-reactive protein (CRP) levels, and other laboratory parameters in patients with UC and CD and with endoscopic disease activity in UC. UC and CD showed comparable areas under the curve (AUC) values for determining clinical remission and differentiating between endoscopic remission associated with LRG and CRP. The levels of LRG mRNA were also increased in PBMCs from patients with UC and CD and reflected disease activity. These data suggest that serum LRG, originated partially from PBMCs, is an inflammatory marker in UC and CD. A large-scale well-designed study should be conducted in the future to more accurately reveal the clinical significance of LRG in patients with IBD.

## 1. Introduction

Inflammatory bowel diseases (IBD), consisting of ulcerative colitis (UC) and Crohn's disease (CD), are chronic disorders involving the gastrointestinal tract. The infiltration and activation of inflammatory cells, and the production of a wide range of mediators, play significant roles in IBD [1, 2].

A reliable surrogate marker capable of mirroring intestinal inflammation and serving as a substitute for endoscopy is required. Blood-based biomarkers typically provide a noninvasive estimation of the inflammatory burden in IBD. However, relatively few blood-based biomarkers have been extensively validated in IBD, and fewer still are in routine use in the clinical setting [2–5]. One of the possible markers is C-reactive protein (CRP). Its clinical level depends on the intensity of the pathological activity, which stimulates CRP production [6]. However, some patients do not develop high CRP levels, in spite of active disease. Therefore, biomarkers of greater sensitivity are warranted.

Leucine-rich alpha-2 glycoprotein (LRG), a 50 kDa glycoprotein that contains repetitive sequences with a leucine-rich motif, was originally identified as an inflammatory biomarker for immune-mediated diseases, such as rheumatoid arthritis and IBD [7]. Subsequent studies reported that LRG levels are increased in other inflammatory diseases, such as Still's disease [8]; Kawasaki disease [9]; juvenile idiopathic arthritis [10]; psoriasis [11]; appendicitis [12]; malignant diseases, such as gastric cancer [13] and colorectal cancer [14]; heart failure [15]; diabetes [16, 17]; and obesity [18]. It is derived predominantly from the neutrophils, macrophages, intestinal epithelial cells, and hepatocytes in response to tumor necrosis factor- (TNF-) *α*, interleukin- (IL-) 1*β*, IL-6, and IL-22 [19].

With respect to UC, the first report on LRG showed that the serum LRG level correlated well with clinical disease activity in 82 patients [19], and a subsequent report from the same laboratory showed that LRG was useful for detecting endoscopic mucosal healing in 129 patients; LRG served as a surrogate marker of endoscopic inflammation [20]. With respect to CD, the first preliminary report from the same group showed that LRG reflected clinical disease activity in 22 patients [7].

To the best of our knowledge, no papers have been published concerning the LRG profile in IBD from a different laboratory, and the clinical significance of LRG measurement is less well established for CD than for UC. Therefore, this study is aimed at comparing the association between serum LRG and CRP levels and clinical and endoscopic disease activity in patients with UC and CD. To the best of our knowledge, this is the first study to evaluate the LRG gene expression in peripheral blood mononuclear cells (PBMCs) in patients with IBD from a different cohort of patients.

## 2. Materials and Methods

### 2.1. Ethical Consideration

The study protocol was reviewed and approved by the Ethics Committee of Kurume University School of Medicine (ID 13098, ID 14253). Informed consent was obtained, before enrollment, from each of the study participants or their parents.

### 2.2. Patients

Between July 2016 and April 2018, serum samples were collected from 98 patients with UC and 96 patients with CD. The pathological diagnoses were based on the characteristic clinical, endoscopic, radiological, and histological features. Ninety-two age-matched individuals served as healthy controls.  Table 1 and Table S1 display the baseline characteristics of the study population.

### 2.3. Evaluation of Disease Activity

For the evaluation of disease, clinical activity was graded using the partial Mayo score (PMS) in patients with UC (inactive disease was defined as a score ≤ 2 with no individual subscore > 1 point) [21] and the Harvey-Bradshaw index (HBI) in patients with CD (inactive disease was defined as a score < 5 points) [22].

The endoscopic activity was graded using the Mayo endoscopic subscore (MES) in patients with UC (endoscopic remission was defined as a score ≤ 1 point) [21] and the simple endoscopic score for Crohn's disease (SES-CD) in patients with CD (endoscopic remission was defined as a score ≤ 1 point) [23]. Blood sampling and colonoscopy were performed on the same day.

### 2.4. Determination of Laboratory Parameters

A blood sample was obtained from each patient to measure various laboratory parameters. The platelet count and serum levels of hemoglobin and albumin were determined by routine laboratory analysis. CRP was determined using latex turbidimetry (FUJIFILM Wako Pure Chemical Corp., Osaka, Japan). The serum LRG levels were quantified using an ELISA (IBL, Fujioka, Japan).

### 2.5. Measurement of LRG mRNA Expression Using Real-Time Quantitative Polymerase Chain Reaction (Real-Time qPCR)

Using PBMCs obtained from a different cohort of patients with IBD (Table S1) [24], we evaluated LRG mRNA levels in patients with IBD. Blood samples (10 mL) were obtained by cubital venous puncture and collected in standard sterile polystyrene vacuum tubes with heparin. First, freshly drawn blood was diluted at a ratio of 1 : 2.5 with phosphate-buffered saline. PBMCs were isolated from the diluted blood by Ficoll-Paque (GE Healthcare, Uppsala, Sweden) density-gradient centrifugation according to the manufacturer's instructions. PBMCs were pelleted, snap-frozen on dry ice, and stored at −80°C until use [25]. RNA was extracted from PBMC samples following the protocol described for the TRIzol Reagent (Invitrogen, Carlsbad, CA, USA). Quantity and purity of the RNA were determined for all the samples on a Nanodrop ND-1000 spectrophotometer (Thermo Scientific, Waltham, MA, USA). The average yield was 23,000 ng. The purity, as measured by the A260/280 ratio, was between 1.91 and 1.95.

Total RNA was converted into cDNA using the ReverTra Ace qPCR RT Kit (Toyobo, Osaka, Japan). The generated cDNAs (25 ng) were stored at −20°C. cDNA was added to the TaqMan Gene Expression Master Mix (Applied Biosystems, CA, USA). qPCR reactions (20 *μ*L) composed of 2 *μ*L cDNA template, TaqMan Universal PCR Master Mix (2x, Thermo Fisher Scientific), TaqMan assay (20x, Thermo Fisher Scientific), and H_2_O, as well as RT-PCR, were performed using the StepOne Real-Time PCR System (Applied Biosystems). Reactions, run in triplicate, were incubated at 50°C for 2 min and 95°C for 10 min, followed by 40 cycles of 95°C for 15 s and 60°C for 1 min. Real-time RT-PCR for *LRG* and *GAPDH* was performed using a TaqMan probe and primer sets for the target as chosen from an online catalog. The reference numbers were as follows: Hs00364835_m1 for *LRG1* and Hs02786624_g1 for *GAPDH* (Applied Biosystems).

### 2.6. Statistical Analysis

Statistical analysis was performed with GraphPad Prism Software (GraphPad Software, San Diego, CA) or IBM SPSS Statistics 23.0 software (IBM, New York). All values are expressed as medians with interquartile ranges (IQR). Differences between groups were compared using the Mann-Whitney *U*-test. Correlations were calculated using the Spearman rank correlation coefficient. Receiver operating characteristic (ROC) curve analysis was performed to assess the diagnostic accuracy of the assay. The cutoff value was determined using the optimal decision threshold. *p* values < 0.05 were considered statistically significant.

## 3. Results

### 3.1. Serum LRG Levels

**Figure 1:**
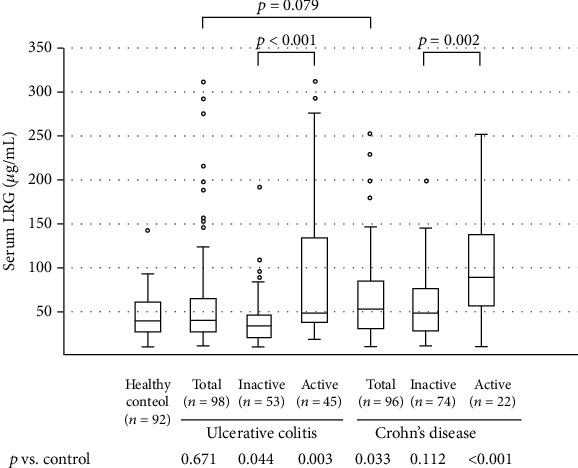
Serum levels of leucine-rich alpha-2 glycoprotein (LRG) in patients with ulcerative colitis, patients with Crohn's disease, and healthy control individuals. The bars indicate the median ± 25^th^ percentile. The lower bar indicates the 10^th^ percentile, and the upper bar indicates the 90^th^ percentile.

Figure 1 shows the serum levels of LRG in patients with UC, patients with CD, and healthy controls. The median (IQR) level of serum LRG (*μ*g/mL) was 38.88 (27.15–59.29) in the healthy controls, 32.27 (20.27–46.66) in patients with inactive UC, 48.85 (35.31–133.77) in patients with active UC, 47.95 (26.97–75.25) in patients with inactive CD, and 89.08 (55.47–136.32) in patients with active CD. No significant differences were found in LRG levels between patients with UC and those with CD. Patients with active UC had higher levels of LRG than did patients with inactive UC (*p* < 0.001) and healthy controls (*p* = 0.003). Patients with active CD had higher levels of LRG than did patients with inactive CD (*p* = 0.002) and healthy controls (*p* < 0.001).

**Figure 2:**
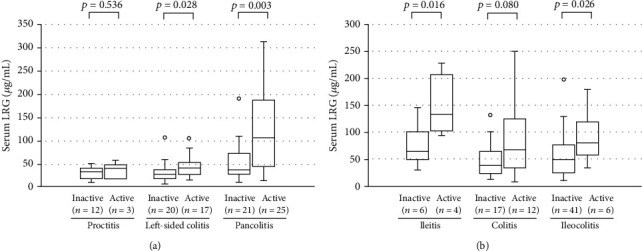
Comparison of the serum leucine-rich alpha-2 glycoprotein (LRG) levels between active and inactive disease, according to the involved area in patients with ulcerative colitis (a) and Crohn's disease (b). The bars indicate the median ± 25^th^ percentile. The lower bar indicates the 10^th^ percentile, and the upper bar indicates the 90^th^ percentile. I: inactive; A: active; n.s.: not significant.

The LRG levels in patients with active disease were also compared according to the disease location (Figure 2). In UC (a), LRG levels were higher in active disease with left-sided (*p* = 0.028) and pancolitis involvement (*p* = 0.003). In CD (Figure 2(b)), LRG levels were higher in active disease with ileitis (*p* = 0.016) and ileocolitis involvement (*p* = 0.026).

### 3.2. Relation to Laboratory Parameters

Table 2 summarizes the correlation coefficients and significance values for LRG levels and the indicated laboratory parameters. LRG levels were significantly correlated with CRP (*r* = 0.647, *p* < 0.001) and serum albumin (*r* = −0.490, *p* < 0.001) in UC and with CRP (*r* = 0.627, *p* < 0.001), serum albumin (*r* = −0.556, *p* < 0.001), and hemoglobin (*r* = −0.407, *p* < 0.001) in CD.

### 3.3. Relation to Clinical Disease Activity

**Figure 3:**
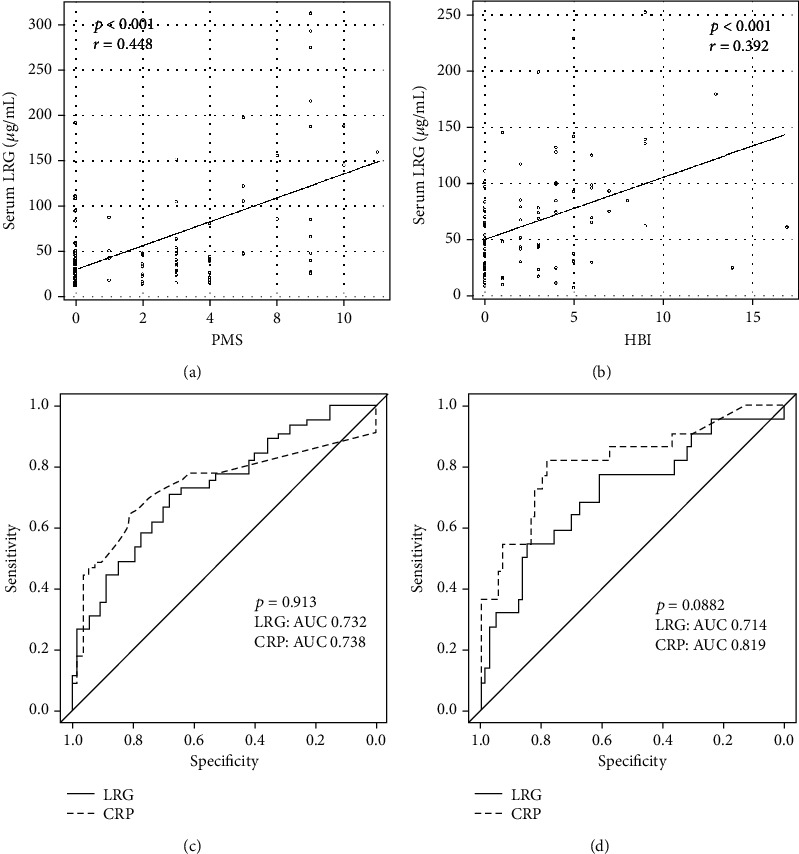
Efficacy of the serum leucine-rich alpha-2 glycoprotein (LRG) level as a biomarker for ulcerative colitis, assessed using the partial Mayo score (PMS), (a) and for Crohn's disease assessed using the Harvey-Bradshaw index (HBI) (b). Receiver operating characteristic (ROC) curves for the serum LRG and C-reactive protein (CRP) levels, as evaluated according to the MS in ulcerative colitis (c) and the HBI in Crohn's disease (d). Values of the area under the ROC curve (AUC) are shown. Clinical remission was defined as MS ≤ 2 in UC and HBI < 5 in CD.

We analyzed the correlation between LRG levels and clinical disease activity. A statistical correlation was observed between LRG levels and PMS in UC (*r* = 0.448, *p* < 0.001; Figure 3(a)) and HBI in CD (*r* = 0.392, *p* < 0.001; Figure 3(b)). This suggests that LRG is a useful marker for evaluating clinical disease activity both in UC and CD.

To assess the diagnostic accuracy of LRG and CRP in evaluating clinical disease activity, we used ROC curve analysis. In the analysis of 98 patients with UC (Figure 3(c)), the area under the curve (AUC) for LRG was 0.732 (95% confidence interval (CI): 0.633–0.831) and 0.738 (0.631–0.844), with a comparable AUC (*p* = 0.913). For identifying clinical remission, the cutoff value for LRG (39.8 *μ*g/mL) had a sensitivity of 71.1% and a specificity of 67.9%, while that for CRP (0.08 mg/dL) had a sensitivity of 71.1% and a specificity of 73.6%. In 96 patients with CD (Figure 3(d)), the AUC values for LRG and CRP were 0.716 (0.584–0.849) and 0.819 (0.708–0.930), respectively; thus, the AUC was comparable (*p* = 0.0882). The cutoff value for LRG (61.3 *μ*g/mL) had a sensitivity of 77.3% and a specificity of 60.8%, while that for CRP (0.45 mg/dL) had a sensitivity of 81.8% and a specificity of 78.1%.

### 3.4. Serial Measurements of LRG

Figure S1 shows the time course of the serum LRG levels, followed longitudinally in two patients with UC and two patients with CD. Eventually, the disease activity in all patients was controlled with treatment. In these cases, the serum levels of LRG and CRP increased during the acute phase and decreased gradually as patients went into remission, regardless of the treatment modality used.

### 3.5. Relation to Endoscopic Disease Activity

**Figure 4:**
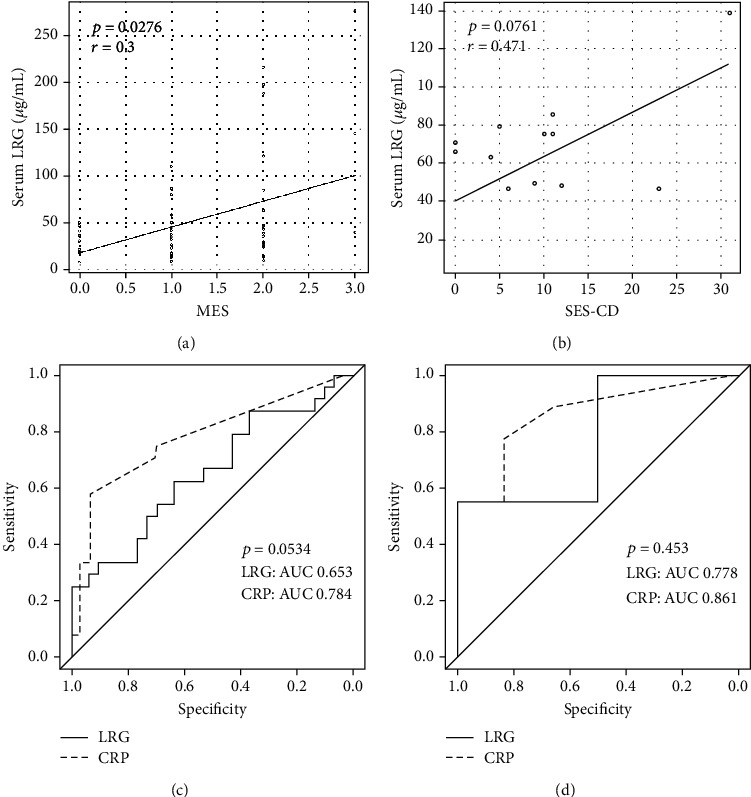
Efficacy of the serum leucine-rich alpha-2 glycoprotein (LRG) level as a biomarker for ulcerative colitis assessed using the Mayo endoscopic subscore (MES) (a) and for Crohn's disease assessed using the simple endoscopic score for Crohn's disease (SES-CD) (b). Receiver operating characteristic (ROC) curves for serum LRG and C-reactive protein (CRP) levels, as evaluated using the MES in ulcerative colitis (c) and the SES-CD in Crohn's disease (d). Values of the area under the ROC curve (AUC) are shown. Endoscopic remission was defined as MES ≤ 1 in UC and SES‐CD ≤ 4 in CD.

We also analyzed the correlation between LRG levels and endoscopic disease activity. A statistical correlation was observed between LRG levels and MES in UC (*r* = 0.3, *p* = 0.0276; Figure 4(a)). A similar trend was observed for the association between LRG levels and SES-CD in CD; however, the correlation was not statistically significant (*r* = 0.471, *p* = 0.0761; Figure 4(b)), suggesting that LRG levels reflect the endoscopic disease activity particularly in UC.

We next compared the diagnostic accuracy of LRG and CRP for evaluating endoscopic disease activity. In 54 patients with UC (Figure 4(c)), the AUC values for LRG and CRP were 0.653 (95% confidence interval: 0.502–0.803) and 0.784 (0.659–0.909), respectively; thus, the AUC values were comparable (*p* = 0.0534). For identifying endoscopic remission, the cutoff value for LRG (40.4 *μ*g/mL) had a sensitivity of 62.5% and a specificity of 63.3%, while that for CRP (0.08 mg/dL) had a sensitivity of 66.7% and a specificity of 80.0%. In 15 patients with CD (Figure 4(d)), the AUC values for LRG and CRP were 0.778 (0.518–1) and 0.861 (0.67–1), respectively; thus, the AUC values were comparable (*p* = 0.453). The cutoff value for LRG (75.2 *μ*g/mL) had a sensitivity of 55.6% and a specificity of 100%, while that for CRP (0.26 mg/dL) had a sensitivity of 77.8% and a specificity of 83.3%.

### 3.6. LRG Gene Expression in PBMCs

**Figure 5:**
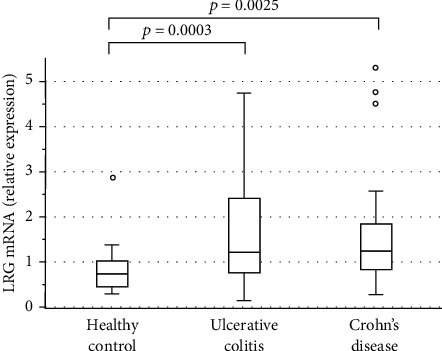
Leucine-rich alpha-2 glycoprotein (LRG) mRNA levels in peripheral blood mononuclear cells obtained from patients with ulcerative colitis, patients with Crohn's disease, and healthy control participants. The bars indicate the median ± 25^th^ percentile. The lower bar indicates the 10^th^ percentile, and the upper bar indicates the 90^th^ percentile.

Using a different cohort of participants, we further examined the LRG gene expression in PBMCs from the patients with IBD and healthy controls using real-time qPCR (Tables S2 and S3). As shown in Figure 5, the LRG mRNA levels were significantly higher both in patients with UC (*p* = 0.0003) and in patients with CD (*p* = 0.0025) compared with those in healthy controls. Table S4 summarizes the correlation coefficients and significance values for the comparisons between LRG mRNA expression levels and clinical disease activity and the indicated laboratory parameters. The LRG mRNA levels were significantly correlated with the PMS in UC (*r* = 0.5062, *p* = 0.0007) and the Crohn's disease activity index (CDAI) in CD (*r* = 0.4859, *p* = 0.0056) [26]. Furthermore, the LRG mRNA levels were positively correlated with the CRP levels both in UC (*r* = 0.4218, *p* = 0.0162) and CD (*r* = 0.4996, *p* = 0.0036) and were negatively correlated with serum albumin and hemoglobin levels only in UC (*r* = −0.6121, *p* = 0.0003 and *r* = −0.4151, *p* = 0.0163, respectively). The serum LRG level was not checked in the same sample since this study was not designed to investigate the LRG profiles.

### 3.7. Effects of Anti-TNF-*α* Treatment on LRG Level and Expression

**Figure 6:**
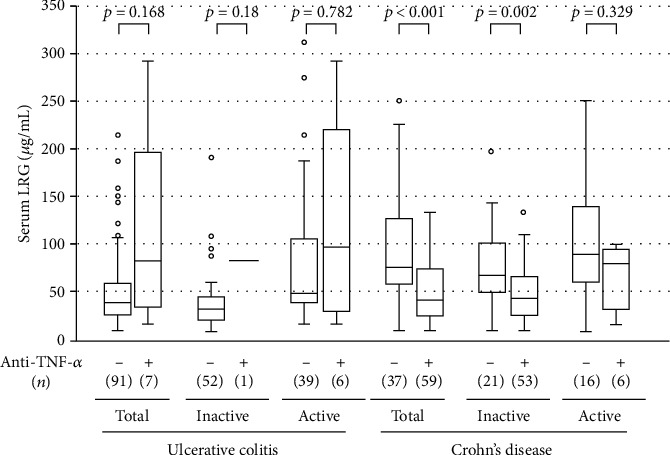
Effect of antitumor necrosis factor- (TNF-) *α* agents on serum leucine-rich alpha-2 glycoprotein (LRG) levels in the patients with ulcerative colitis and those with Crohn's disease. For each disease, patients were divided into two treatment-based subgroups: patients taking anti-TNF-*α* agents and patients receiving any other medication. The bars indicate the median ± 25^th^ percentile. The lower bar indicates the 10^th^ percentile, and the upper bar indicates the 90^th^ percentile.

Patients with IBD take a variety of medications, including biologics, which may be linked to alterations in LRG levels. Among patients with CD, serum LRG levels showed a trend toward lower levels in those with anti-TNF-*α* treatment (*n* = 59) than in those without anti-TNF-*α* treatment (*n* = 37) (Figure 6); additionally, in those with anti-TNF-*α* treatment, the LRG level was correlated with several laboratory and disease activity markers (Table S5). Among patients with UC, serum LRG levels were comparable between those with anti-TNF-*α* treatment (*n* = 7) and without anti-TNF-*α* treatment (*n* = 91), although, the number of patients receiving anti-TNF-*α* agents was very small (Figure 6); additionally, among those with anti-TNF-*α* treatment, the LRG level was correlated with several laboratory and disease activity markers (Table S5). Furthermore, among patients with UC, there were no significant differences in LRG mRNA in PBMCs between those with and without anti-TNF-*α* treatment (*n* = 3*vs.* 38, respectively); among patients with CD (*n* = 24*vs.* 10, respectively), LRG expression tended to be lower in those with anti-TNF-*α* treatment than in those without anti-TNF-*α* treatment (Figure S2).

## 4. Discussion

There are several reports concerning biomarkers for intestinal inflammation [2, 4, 5, 27, 28]. However, the role of serum LRG in IBD is not clearly known. In the present study, we examined the serum level of LRG from patients with IBD and evaluated its relationship with clinical, endoscopic, and laboratory parameters. The aim of the present study was to evaluate the utility of serum LRG as a potential inflammatory marker for IBD and to investigate LRG gene expression in PBMCs as a possible source of serum LRG.

The present study showed that the LRG levels were higher in CD and UC patients than in healthy controls. In patients with UC, the LRG levels were correlated with laboratory parameters and clinical disease activity. The results of our study are similar to the findings of Serada et al. [19]. Our study also revealed a significant correlation between LRG and UC endoscopic disease activity, similar to the report by Shinzaki et al. [20]. Given the potential role of LRG as a biomarker for clinical and endoscopic disease activity in UC, we further investigated its diagnostic accuracy for detecting endoscopic remission and found that the AUC was similar for LRG and CRP, which differed from previous findings that showed a higher AUC for LRG than for CRP [20], although the diagnostic criteria of endoscopic remission were different. Additional studies are warranted to evaluate the clinical significance of LRG measurement in UC.

Compared with UC, the clinical significance of LRG measurement in CD has been less well evaluated. Serada et al. preliminarily measured LRG levels in 22 patients with CD and found that LRG was significantly correlated with clinical disease activity but not CRP [7]. In the present study, we evaluated LRG levels in 96 patients with CD and found that LRG correlated with clinical disease activity, as well as with CRP levels. Furthermore, we assessed, for the first time in CD, endoscopic disease activity in relation to LRG levels and found that LRG tended to correlate with endoscopic disease activity as assessed by SES-CD, but the correlation did not reach statistical significance. One possible explanation for this discrepancy is that the SES-CD, which includes the assessment of colonoscopy alone, was insufficient to evaluate the activity of an entire small intestinal lesion. In fact, this study included 27 of 34 patients with small bowel-involved CD. It is not easy to evaluate small bowel inflammation because an appropriate diagnostic method has not been validated for assessing small bowel CD. Another explanation was the small number of patients included in this analysis since we limited it to patients with CD who received blood sampling and colonoscopy on the same day. Further studies are needed on the significance of serum LRG as a biomarker for the screening of small and large bowel inflammation in CD.

A serial change in clinical disease activity, serum CRP, and LRG in both patients with UC and CD observed in the present study seems to be related, indicating the suitability of serum LRG as a monitoring tool in the same patient regardless of the treatment modality. Ultimately, our longitudinal study showed that serum LRG was detectable in every patient, even when CRP was undetectable. Although future studies are needed, these observations suggest that serum LRG could provide more relevant information on inflammatory response than CRP. Implementing treat-to-target strategies involves a regular assessment of objective markers of disease activity and the adjustment of therapy when needed.

The precise origin of increased circulating LRG in IBD remains unclear. Western blotting analysis demonstrated that LRG is upregulated markedly in inflamed colonic tissues [19], indicating that it is predominantly derived from the swollen intestine. In the present study, we demonstrated for the first time that LRG gene expression in PBMCs from UC and CD is also increased and reflects the clinical disease activity, indicating that increased serum LRG observed in IBD is at least in part originating from PBMCs. Previous studies showed that LRG is produced by neutrophils, macrophages, hepatocytes [29], and intestinal epithelial cells in response to IL-1*β*, IL-6, TNF-*α*, and IL-22, suggesting the involvement of an IL-6-independent pathway [30]. Taken together, serum LRG would appear to be derived from peripheral leukocytes in response to stimuli in the circulation, as well as the liver and diseased intestine. This is in contrast to CRP, which is a serum acute phase reactant protein of hepatic origin, in response to IL-6 released from macrophages and lymphocytes in the diseased intestine [31–33]. These varied origins of serum LRG and CRP may be complementary for the accurate evaluation of disease activity.

Since TNF-*α*, in addition to IL-1*β*, IL-6, and IL-22, is involved in LRG production *in vitro* [29], we further evaluated the effect of anti-TNF-*α* treatment on serum LRG levels. We found that serum LRG was lower in those under anti-TNF-*α* treatment than in those not under anti-TNF-*α* treatment, particularly in CD, suggesting that the anti-TNF-*α* agent could in itself affect LRG levels. Importantly, it was also shown that the serum LRG is correlated with laboratory and disease activity parameters. These results indicate that serum LRG is a potential inflammatory biomarker regardless of the use of anti-TNF-*α* agents. This was further supported by the present results pertaining to serial changes in the serum LRG level, which was associated with CRP and disease activity in patients under anti-TNF-*α* treatment. However, as only limited data are available at present, further studies on the effect of anti-TNF-*α* agents on serum LRG levels are warranted.

Our study has some limitations. First, this study was performed as a single-center analysis and involved a limited number of participants. It is crucial to increase the number of studies and participants involved. Second, the number of patients who underwent colonoscopy during the observation period was limited relative to the number of patients studied; therefore, it is difficult to evaluate the superiority of LRG to CRP in reflecting endoscopic remission. Third, it is important to point out that many of our patients were receiving medical treatment at the time they were evaluated. Finally, since we used PBMCs from patients with IBD from a previous different project, we could not examine the direct correlation between serum LRG protein and PBMC LRG mRNA in the same sample. However, the present study focused on exploring the serum LRG profile in patients with IBD.

## 5. Conclusions

In conclusion, this is the first study to evaluate the usefulness of serum LRG as an IBD biomarker by a research group other than the group that initially conducted research into the development of LRG as a biomarker and revealed that serum LRG reflects IBD disease activity and has potential in playing an important role in treat-to-target strategies. Moreover, we demonstrated for the first time that LRG mRNA in PBMCs reflects IBD disease activity, suggesting that serum LRG is, at least in part, derived from PBMCs. A future, large-scale, well-designed study could more accurately reveal the clinical significance of LRG in patients with IBD.

## Figures and Tables

**Table 1:** Baseline characteristics of the study population used to evaluate LRG concentrations in the sera of patients with IBD and healthy individuals.

	Ulcerative colitis	Crohn's disease	Healthy individuals
No. of participants	98	96	92
Sex (male/female)	54/44	54/42	52/40
Age (years) (median, IQR)	41.6 (33.0–59.7)	33.5 (24.3–48.9)	39.6 (30.4–49.6)
Disease distribution	Proctitis/left-sided colitis/pancolitis 15/37/46	Ileitis/colitis/ileocolitis 20/29/47	—
Disease duration (years) (median, IQR)	4.37 (1.0–9.0)	9.1 (2.91–15.8)	—
Treatments			
5-Aminosalicylic acid (%)	Oral 72 (73.4), topical 26 (27.0)	Oral 76 (79.2)	—
Prednisolone (%)	Oral 17 (17.3), topical 8 (8.1)	Oral 14 (14.6)	—
Immunomodulator (%)	22 (22.4)	35 (37.1)	—
Leukocytapheresis (%)	6 (6.1)	1 (1.0)	—
Antitumor necrosis factor-*α* (%)	7 (7.1)	59 (61.5)	—
Indigo naturalis (%)	19 (19.3)	0 (0)	—
None (%)	9 (9.1)	9 (9.3)	—

LRG: leucine-rich alpha-2 glycoprotein; IBD: inflammatory bowel disease; IQR: interquartile range.

**Table 2:** Correlation coefficients and significance of differences in the levels of serum LRG and laboratory parameters between patients with ulcerative colitis and Crohn's disease.

	Ulcerative colitis		Crohn's disease	
	*r*	*p*	*r*	*p*
Hemoglobin	-0.174	0.086	-0.407	<0.001
Albumin	-0.490	<0.001	-0.556	<0.001
CRP	0.647	<0.001	0.627	<0.001

LRG: leucine-rich alpha-2 glycoprotein; CRP: C-reactive protein. Correlation analysis was performed using Spearman's rank correlation test.

## Data Availability

The datasets during and/or analyzed during the current study are available from the corresponding author on reasonable request.

## Supplementary Materials

Supplementary 1 Figure S1: time courses for serum leucine-rich alpha-2 glycoprotein (LRG), serum C-reactive protein (CRP), and disease activity levels in four patients with inflammatory bowel disease. The serum samples were obtained during the active and inactive stages of the disease in two patients with ulcerative colitis (UC) and two patients with Crohn's disease (CD). The detection limit of CRP was <0.05 mg/dL. A: a 28-year-old man with left-sided UC was treated with 5-ASA, PSL, and AZA. B: a 37-year-old woman with left-sided UC was treated with 5-ASA and PSL. C: a 26-year-old man with ileitis due to CD was treated with AZA and anti-TNF-*α*. D: a 21-year-old woman with ileocolitis due to CD was treated with 5-ASA, PSL, and anti-TNF-*α*. The clinical activity was assessed using the partial Mayo score for patients with UC and the Harvey-Bradshaw index for patients with CD. 5-ASA: 5-aminosalicylic acid; TNF-*α*: tumor necrosis factor-*α*; PSL: prednisolone; AZA: azathioprine. Figure S2: effect of antitumor necrosis factor- (TNF-) *α* agents on leucine-rich alpha-2 glycoprotein (LRG) mRNA levels in peripheral blood mononuclear cells obtained from patients with ulcerative colitis and Crohn's disease. For each disease, patients were divided into two treatment-based subgroups: patients taking anti-TNF-*α* agents and patients receiving any other medication. The bars indicate the median ±25^th^ percentile. The lower bar indicates the 10^th^ percentile, and the upper bar indicates the 90^th^ percentile.

Supplementary 2 Table S1: baseline characteristics of the study population for the serum LRG concentration assessment. LRG: leucine-rich alpha-2 glycoprotein; IBD: inflammatory bowel disease; CRP: C-reactive protein; PMS: partial Mayo score; HBI: Harvey-Bradshaw index; MES: Mayo endoscopic subscore; SESCD: simple endoscopic score for Crohn's disease. Data are expressed as the median (IQR (interquartile range)). Table S2: baseline characteristics of the study population for the PBMC LRG mRNA assessment. LRG: leucine-rich alpha-2 glycoprotein; PBMCs: peripheral blood mononuclear cells; IBD: inflammatory bowel disease; IQR: interquartile range. Table S3: baseline characteristics of the study population for the PBMC LRG mRNA assessment. LRG: leucine-rich alpha-2 glycoprotein; PBMCs: peripheral blood mononuclear cells; IBD: inflammatory bowel disease; CRP: C-reactive protein; PMS: partial Mayo score; CDAI: Crohn's disease activity index. Data are expressed as the median (IQR (interquartile range)). Table S4: correlation coefficients and significance of the differences between PBMC LRG mRNA levels and clinical disease activities and laboratory parameters in patients with ulcerative colitis and Crohn's disease. PBMCs: peripheral blood mononuclear cells; LRG: leucine-rich alpha-2 glycoprotein; PMS: partial Mayo score; CDAI: Crohn's disease activity index; CRP: C-reactive protein. Correlation analysis was performed using Spearman's rank correlation test. Table S5: effect of antitumor necrosis factor- (TNF-) *α* agents on the correlation coefficient and significance of the differences between the serum LRG level and laboratory parameters in patients with ulcerative colitis and Crohn's disease. For each disease, patients were divided into two treatment-based subgroups: patients taking anti-TNF-*α* agents and patients receiving any other medication. Clinical disease activity was assessed using the partial Mayo score (PMS) for ulcerative colitis and the Harvey-Bradshaw index (HBI) for Crohn's disease. Endoscopic disease activity was assessed using the Mayo endoscopic subscore (MES) for ulcerative colitis and the simple endoscopic score for Crohn's disease (SES-CD) for Crohn's disease. Correlational analysis was performed using Spearman's rank correlation test. TNF: tumor necrosis factor; CRP: C-reactive protein.
