# Supplementary figures and images for: Evaluation of Serum Leucine-Rich Alpha-2 Glycoprotein as a New Inflammatory Biomarker of Inflammatory Bowel Disease
Source: Mediators Inflamm. 2021 Feb 1;2021:8825374. doi: 10.1155/2021/8825374 (PMC7874844; doi:10.1155/2021/8825374)

Figure S1

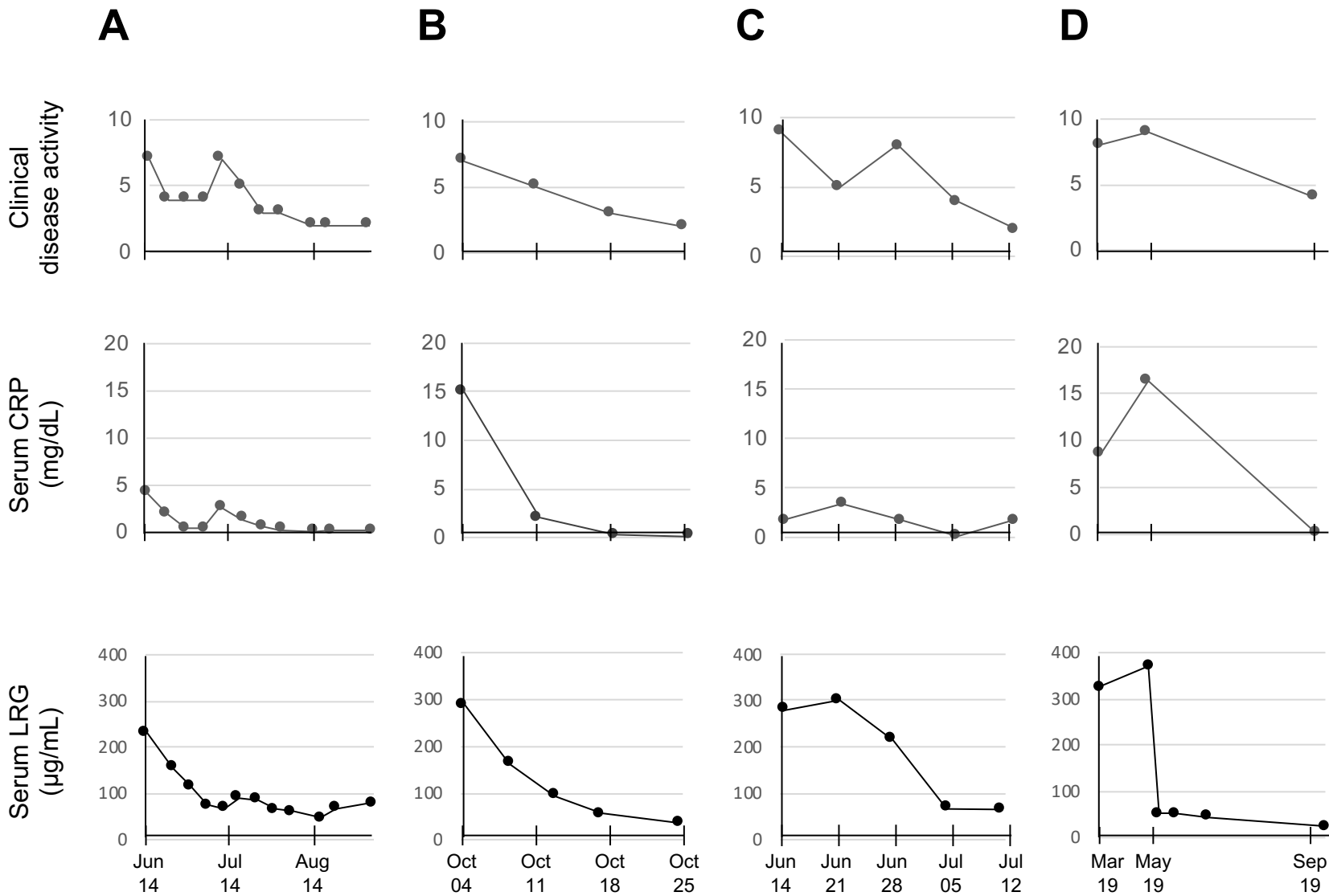

Supplement: Supplementary 1 — Figure S1: time courses for serum leucine-rich alpha-2 glycoprotein (LRG), serum C-reactive protein (CRP), and disease activity levels in four patients with inflammatory bowel disease. The serum samples were obtained during the active and inactive stages of the disease in two patients with ulcerative colitis (UC) and two patients with Crohn's disease (CD). The detection limit of CRP was <0.05 mg/dL. A: a 28-year-old man with left-sided UC was treated with 5-ASA, PSL, and AZA. B: a 37-year-old woman with left-sided UC was treated with 5-ASA and PSL. C: a 26-year-old man with ileitis due to CD was treated with AZA and anti-TNF-α. D: a 21-year-old woman with ileocolitis due to CD was treated with 5-ASA, PSL, and anti-TNF-α. The clinical activity was assessed using the partial Mayo score for patients with UC and the Harvey-Bradshaw index for patients with CD. 5-ASA: 5-aminosalicylic acid; TNF-α: tumor necrosis factor-α; PSL: prednisolone; AZA: azathioprine. Figure S2: effect of antitumor necrosis factor- (TNF-) α agents on leucine-rich alpha-2 glycoprotein (LRG) mRNA levels in peripheral blood mononuclear cells obtained from patients with ulcerative colitis and Crohn's disease. For each disease, patients were divided into two treatment-based subgroups: patients taking anti-TNF-α agents and patients receiving any other medication. The bars indicate the median ±25th percentile. The lower bar indicates the 10th percentile, and the upper bar indicates the 90th percentile. [file 8825374.f1.pdf]
