# Supplementary material for: Evaluation of Serum Leucine-Rich Alpha-2 Glycoprotein as a New Inflammatory Biomarker of Inflammatory Bowel Disease
Source: Mediators Inflamm. 2021 Feb 1;2021:8825374. doi: 10.1155/2021/8825374 (PMC7874844; doi:10.1155/2021/8825374)

Table S1. Baseline characteristics of the study population for the serum LRG concentration assessment

|  | **Ulcerative colitis** (n=98) | **Crohn’s disease** (n=96) |
| --- | --- | --- |
| Hemoglobin (g/dl) | 13.4 (11.88-14.63) | 12.7 (10.6-14.7) |
| Albumin (g/dl) | 4.27 (4.00-4.54) | 4.17 (3.61-4.43) |
| CRP (mg/dl) | 0.07 (0.04-0.19) | 0.18 (0.04-0.8) |
| LRG (μg/mL) | 39.39 (26.59-64.95) | 51.66 (29.36-84.68) |
| Clinical activity index |  |  |
| PMS | 2 (0-4) | — |
| HBI | — | 1 (0-4) |
| Endoscopic activity index |  |  |
| MES | 1 (1-2) | — |
| SESCD | — | 6 (0-11) |

LRG, leucine-rich alpha-2 glycoprotein; IBD, inflammatory bowel disease; CRP, C-reactive protein; PMS, partial mayo score; HBI, Harvey Bradshaw index; MES, Mayo endoscopic subscore; SESCD, Simple endoscopic score for Crohn’s disease; Data are expressed as median (IQR (interquartile range))

Table S2. Baseline characteristics of the study population for the PBMC LRG mRNA assessment

|  | Ulcerative colitis | Crohn’s disease | Healthy subjects |
| --- | --- | --- | --- |
| No. of Patients | 41 | 34 | 30 |
| Sex (male/female) | 19/22 | 12/12 | 13/17 |
| Age (years)  (median, IQR) | 41  (29-58) | 35  (27-47) | 39  (32-44) |
| Disease distribution | Proctitis/Left-sided colitis/Pancolitis  7/8/26 | Ileitis/Colitis/Ileocolitis  4/7/23 |  |
| Disease duration (months)  (median, IQR) | 81  (60.5-171.5) | 106  (60.9-274.2) |  |
| Treatments |  |  |  |
| 5-aminosalicylic acid (%) | 36 (87.8) | 25 (73.5) |  |
| Prednisolone (%) | 7 (17.1) | 3 (8.8) |  |
| Immunomodulator (%) | 7 (17.1) | 9 (26.5) |  |
| Leukocytapheresis (%) | 2 (4.9) | 0 (0.0) |  |
| Anti-tumor necrosis factor (%) | 3 (7.3) | 24 (70.6) |  |
| None (%) | 2 (4.9) | 0 (0.0) |  |

LRG, leucine-rich alpha-2 glycoprotein; PBMCs, peripheral blood mononuclear cells; IBD; inflammatory bowel disease; IQR, interquartile range

Table S3. Baseline characteristics of the study population for the PBMC LRG mRNA assessment

|  | **Ulcerative colitis** (n=41) | **Crohn’s disease** (n=34) |
| --- | --- | --- |
| Hemoglobin (g/dl) | 13.3 (10.4-14.5) | 12.1 (10.9-14.6) |
| Albumin (g/dl) | 3.93 (3.66-4.25) | 3.93 (3.62-4.37) |
| CRP (mg/dl) | 0.11 (0.04-0.53) | 0.13 (0.04-0.31) |
| Clinical activity index |  |  |
| PMS | 2 (0-5) | — |
| CDAI | — | 142 (62-216) |

LRG, leucine-rich alpha-2 glycoprotein; PBMCs, peripheral blood mononuclear cells; IBD; inflammatory bowel disease; CRP, C-reactive protein; PMS, partial mayo score; CDAI, Crohn’s disease activity index; Data are expressed as median (IQR (interquartile range))

Table S4. Correlation coefficients and significance of differences between the PBMC LRG mRNA levels and clinical disease activities or laboratory parameters in patients with ulcerative colitis and Crohn’s disease

|  | Ulcerative colitis | | Crohn’s disease | |
| --- | --- | --- | --- | --- |
|  | *r* | *p* | *r* | *p* |
| PMS | 0.5062 | 0.0007 | ⎯ | ⎯ |
| CDAI | ⎯ | ⎯ | 0.4859 | 0.0056 |
| Hemoglobin | -0.4151 | 0.0163 | 0.0783 | 0.6701 |
| Albumin | -0.6121 | 0.0003 | -0.2268 | 0.2118 |
| CRP | 0.4218 | 0.0162 | 0.4996 | 0.0036 |

PBMCs, peripheral blood mononuclear cells; LRG, leucine-rich alpha-2 glycoprotein; PMS, partial Mayo score; CDAI, Crohn’s disease activity index; CRP, C-reactive protein

Correlation analysis was performed using Spearman's rank correlation test.

Table S5. Effect of anti-tumor necrosis factor (TNF)-α agents on the correlation coefficient and significance of the differences between the serum LRG level and laboratory parameters in patients with ulcerative colitis and Crohn’s disease. For each disease, patients were divided into two treatment-based subgroups: patients taking anti-TNF-α agents and patients receiving any other medication.

|  | Ulcerative colitis | | | | | | |  | Crohn’s disease | | | | | | |
| --- | --- | --- | --- | --- | --- | --- | --- | --- | --- | --- | --- | --- | --- | --- | --- |
|  | Anti-TNF-α (⎯) | | |  | Anti-TNF-α (+) | | |  | Anti-TNF-α (⎯) | | |  | Anti-TNF-α (+) | | |
|  | *r* | *p* | *n* |  | *r* | *p* | *n* |  | *r* | *p* | *n* |  | *r* | *p* | *n* |
| Hemoglobin | -0.1546 | 0.1432 | 91 |  | -0.3783 | 0.4026 | 7 |  | -0.1523 | 0.368 | 37 |  | -0.37 | 0.0042 | 59 |
| Albumin | -0.441 | <0.0001 | 91 |  | -0.8468 | 0.0162 | 7 |  | -0.2781 | 0.1005 | 37 |  | -0.5336 | <0.0001 | 59 |
| CRP | 0.6221 | <0.0001 | 91 |  | 0.9549 | 0.0008 | 7 |  | 0.4326 | 0.0074 | 37 |  | 0.5861 | <0.0001 | 59 |
| PMS | 0.4459 | <0.0001 | 91 |  | 0.6306 | 0.1289 | 7 |  | ⎯ | ⎯ | ⎯ |  | ⎯ | ⎯ | ⎯ |
| HBI | ⎯ | ⎯ | ⎯ |  | ⎯ | ⎯ | ⎯ |  | 0.1891 | 0.2622 | 37 |  | 0.3413 | 0.0081 | 59 |
| MES | 0.3627 | 0.001 | 77 |  | 0.866 | 0.3333 | 3 |  | ⎯ | ⎯ | ⎯ |  | ⎯ | ⎯ | ⎯ |
| SESCD | ⎯ | ⎯ | ⎯ |  | ⎯ | ⎯ | ⎯ |  | 0.8964 | 0.0062 | 7 |  | 0.4721 | 0.199 | 9 |
|  |  |  |  |  |  |  |  |  |  |  |  |  |  |  |  |

Clinical disease activity was assessed using the partial Mayo score (PMS) for ulcerative colitis and the Harvey-Bradshaw index (HBI) for Crohn’s disease. Endoscopic disease activity was assessed using the Mayo endoscopic subscore (MES) for ulcerative colitis and the simple endoscopic score for Crohn's disease (SESCD) for Crohn’s disease. Correlational analysis was performed using the Spearman's rank correlation test. TNF, tumor necrosis factor; CRP, C-reactive protein.


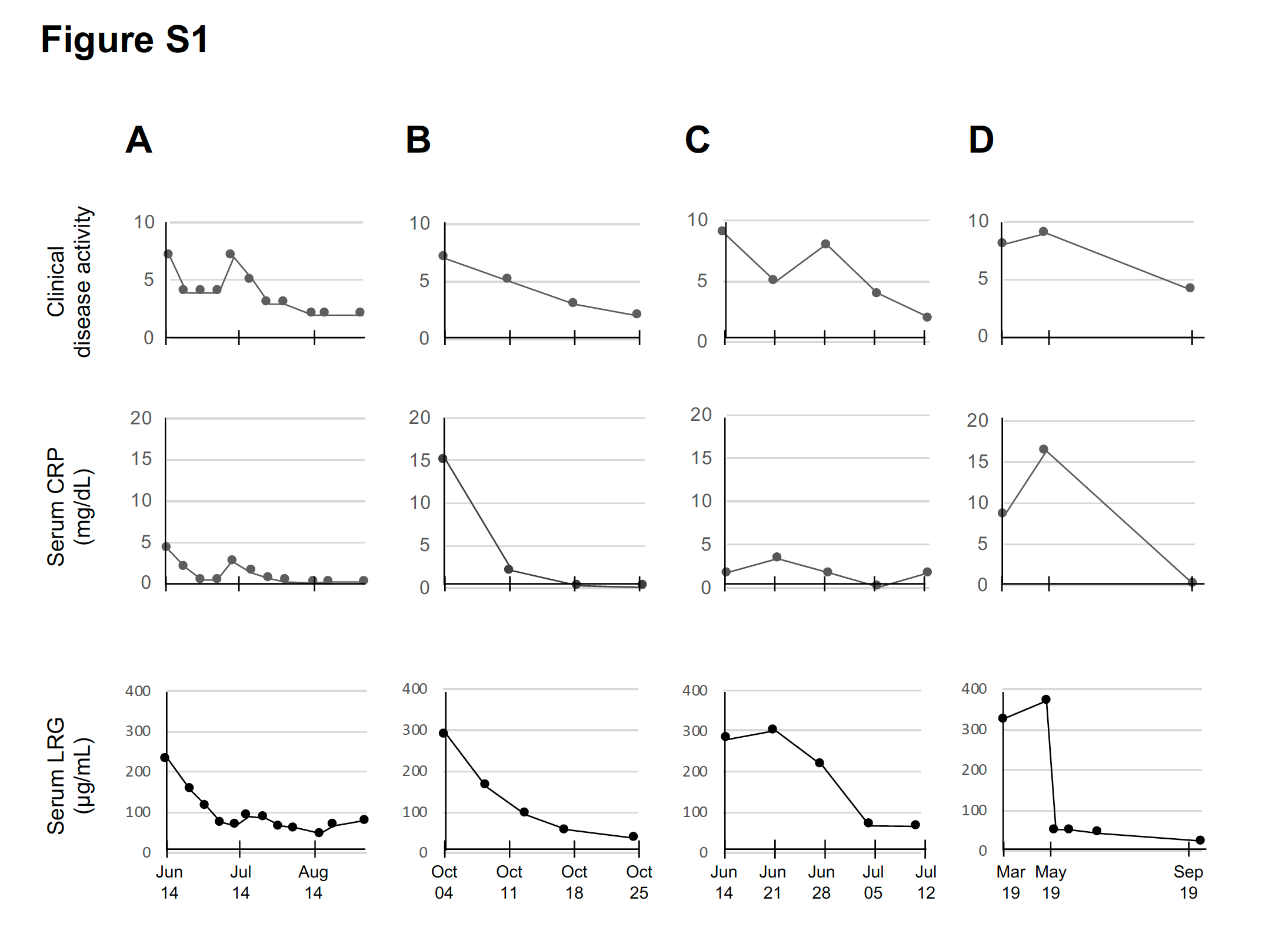


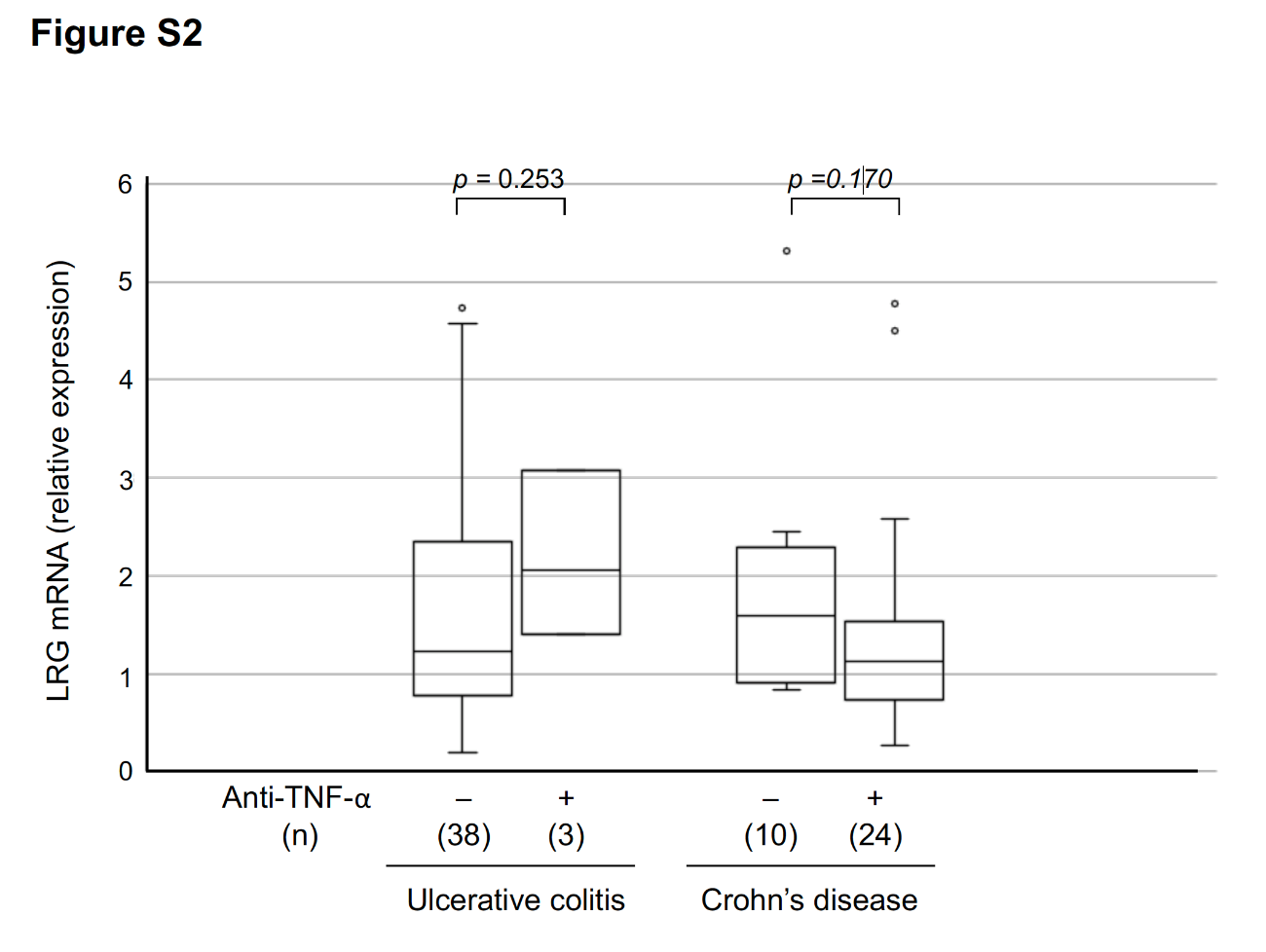

Supplement: Supplementary 2 — Table S1: baseline characteristics of the study population for the serum LRG concentration assessment. LRG: leucine-rich alpha-2 glycoprotein; IBD: inflammatory bowel disease; CRP: C-reactive protein; PMS: partial Mayo score; HBI: Harvey-Bradshaw index; MES: Mayo endoscopic subscore; SESCD: simple endoscopic score for Crohn's disease. Data are expressed as the median (IQR (interquartile range)). Table S2: baseline characteristics of the study population for the PBMC LRG mRNA assessment. LRG: leucine-rich alpha-2 glycoprotein; PBMCs: peripheral blood mononuclear cells; IBD: inflammatory bowel disease; IQR: interquartile range. Table S3: baseline characteristics of the study population for the PBMC LRG mRNA assessment. LRG: leucine-rich alpha-2 glycoprotein; PBMCs: peripheral blood mononuclear cells; IBD: inflammatory bowel disease; CRP: C-reactive protein; PMS: partial Mayo score; CDAI: Crohn's disease activity index. Data are expressed as the median (IQR (interquartile range)). Table S4: correlation coefficients and significance of the differences between PBMC LRG mRNA levels and clinical disease activities and laboratory parameters in patients with ulcerative colitis and Crohn's disease. PBMCs: peripheral blood mononuclear cells; LRG: leucine-rich alpha-2 glycoprotein; PMS: partial Mayo score; CDAI: Crohn's disease activity index; CRP: C-reactive protein. Correlation analysis was performed using Spearman's rank correlation test. Table S5: effect of antitumor necrosis factor- (TNF-) α agents on the correlation coefficient and significance of the differences between the serum LRG level and laboratory parameters in patients with ulcerative colitis and Crohn's disease. For each disease, patients were divided into two treatment-based subgroups: patients taking anti-TNF-α agents and patients receiving any other medication. Clinical disease activity was assessed using the partial Mayo score (PMS) for ulcerative colitis and the Harvey-Bradshaw index (HBI) for Crohn's disea [file 8825374.f2.docx]
